# Recommendations for the management of yttrium-90 radioembolization in the treatment of patients with colorectal cancer liver metastases: a multidisciplinary review

**DOI:** 10.1007/s12094-023-03299-y

**Published:** 2023-09-25

**Authors:** Encarna González-Flores, Natalia Zambudio, Pedro Pardo-Moreno, Beatriz Gonzalez-Astorga, Jorge Roldán de la Rúa, Eva M. Triviño-Ibáñez, Pablo Navarro, Nataly Espinoza-Cámac, Miguel Ángel Casado, Antonio Rodríguez-Fernández

**Affiliations:** 1 Medical Oncology Department, Hospital Universitario Virgen de las Nieves, Granada, Spain; 2 Instituto de Investigación Biosanitaria IBS, Granada, Spain; 3 Surgery Department, Hospital Universitario Virgen de las Nieves, Granada, Spain; 4 Radiodiagnostic Department, Hospital Universitario Virgen de las Nieves, Granada, Spain; 5 Medical Oncology Department, Hospital Universitario Clínico San Cecilio, Granada, Spain; 6 Surgery Department, Hospital Universitario Virgen de la Victoria, Málaga, Spain; 7 Nuclear Medicine Department, Hospital Universitario Virgen de las Nieves, Granada, Spain; 8 Pharmacoeconomics and Outcomes Research Iberia (PORIB), Paseo Joaquín Rodrigo 4-I, Pozuelo de Alarcón, 28224 Madrid, Spain

**Keywords:** Liver metastases, Colon, Radioembolization, Yttrium-90

## Abstract

**Purpose:**

Strategies for the treatment of liver metastases from colon cancer (lmCRC) are constantly evolving. Radioembolization with yttrium 90 (Y-90 TARE) has made significant advancements in treating liver tumors and is now considered a potential option allowing for future resection. This study reviewed the scientific evidence and developed recommendations for using Y-90 TARE as a treatment strategy for patients with unresectable lmCRC.

**Methods:**

A multidisciplinary scientific committee, consisting of experts in medical oncology, hepatobiliary surgery, radiology, and nuclear medicine, all with extensive experience in treating patients with ImCRC with Y-90 TARE, led this project. The committee established the criteria for conducting a comprehensive literature review on Y-90 TARE in the treatment of lmCRC. The data extraction process involved addressing initial preliminary inquiries, which were consolidated into a final set of questions.

**Results:**

This review offers recommendations for treating patients with lmCRC using Y-90 TARE, addressing four areas covering ten common questions: 1) General issues (multidisciplinary tumor committee, indications for treatment, contraindications); 2) Previous process (predictive biomarkers for patient selection, preintervention tests, published evidence); 3) Procedure (standard procedure); and 4) Post-intervention follow-up (potential toxicity and its management, parameters for evaluation, quality of life).

**Conclusions:**

Based on the insights of the multidisciplinary committee, this document offers a comprehensive overview of the technical aspects involved in the management of Y-90 TARE. It synthesizes recommendations for applying Y-90 TARE across various phases of the treatment process.

## Introduction

Colorectal cancer (CRC) is the second leading cause of cancer-related deaths worldwide [1]. It has the highest incidence in Spain, with an estimated 42,721 new cases in projected for 2023 [1]. Approximately 50% of patients with CRC present with metastases at the time of diagnosis (synchronous metastases) or develop them throughout the course of the disease (metachronous metastases), with liver metastases (lmCRC) being the most frequent location [2]. In these cases, surgical resection is an option with curative intent; however, only 10–20% of patients can be considered suitable for resection at onset [3, 4]. The remaining patients may present an unresectable pathology without the possibility of future resection. Alternatively, in a more positive scenario, they could opt for a strategy that improve the conditions for subsequent tumor resection [3, 4]. Radioembolization with yttrium-90 (Y-90 TARE), which consists of irradiating the tumor within the liver parenchyma using ^90^Y microspheres, plays an increasingly relevant role in the therapeutic strategy for treating lmCRC [3, 4]. It is useful in patients who are refractory to lmCRC treatment, and there is growing interest in its use in the early stages of the disease [3, 4].

Given the absence of a specific consensus document on the indications and management of Y-90 TARE in patients with unresectable lmCRC, the objective of this document is to review current scientific evidence and develop general recommendations to enhance understanding of this treatment strategy in routine clinical practice.

## Methodology

**Fig. 1:**
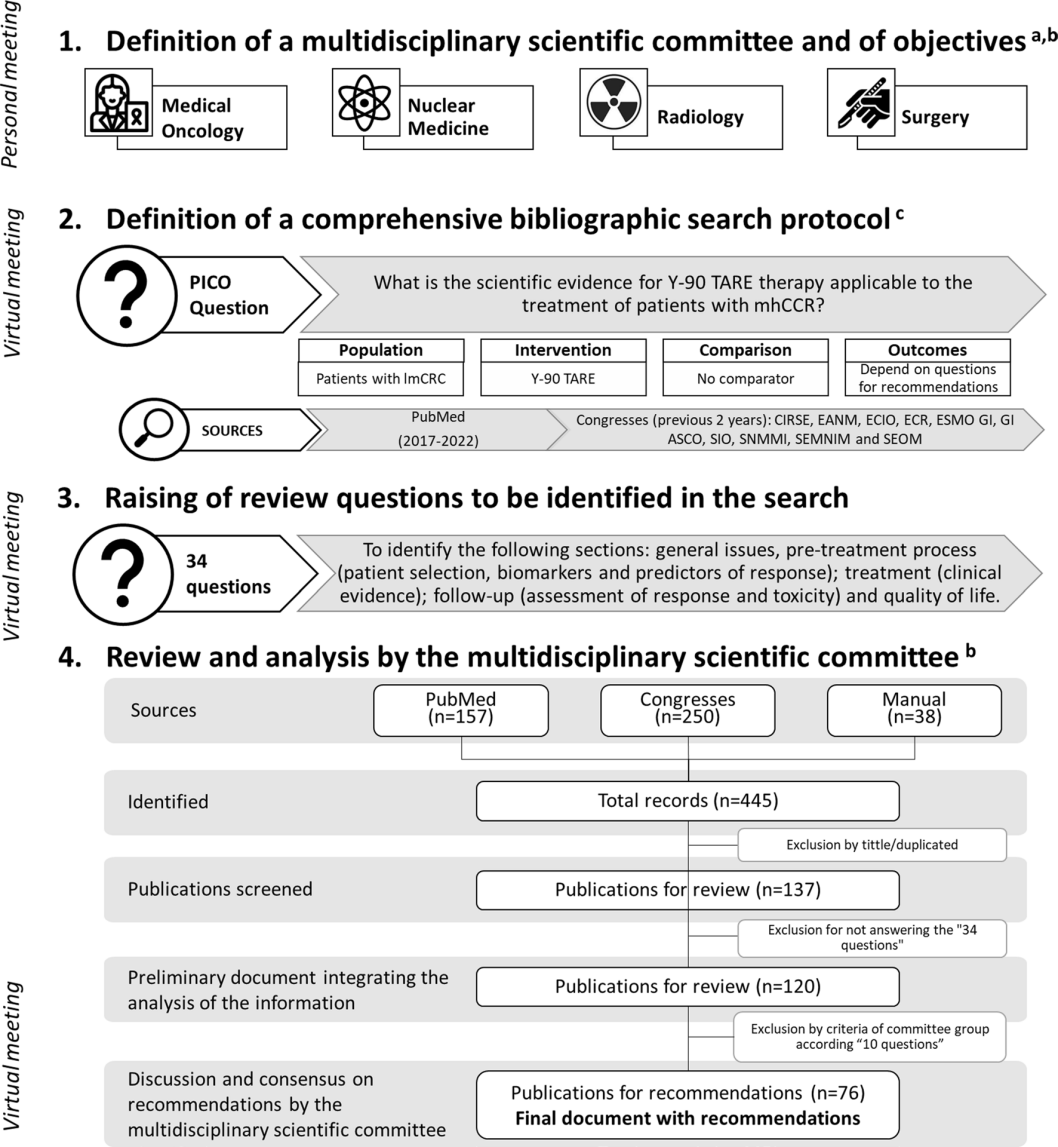
Recommendation development process. **a** Members of the expert group are arranged alphabetically according to the specialty. **b** Members of the expert group met online for joint agreement. The follow-up of the development and validation of the document was done by email. **c** Congresses included within the search protocol: Cardiovascular and Interventional Radiological Society of Europe (CIRSE), European Conference on Interventional Oncology (ECIO), European Association of Nuclear Medicine (EANM), Society of Interventional Oncology (SIO), European Congress of Radiology (ECR), Society of Nuclear Medicine and Molecular Imaging (SNMMI), Gastrointestinal Cancers Symposium of American Society of Clinical Oncology (GI ASCO), World Congress on Gastrointestinal Cancer of European Society for Medical Oncology (ESMO GI), Spanish Society of Nuclear Medicine and Molecular Imaging (SEMNIM) and Spanish Society of Medical Oncology (SEOM)

The project was conducted in two phases involving several deliberative meetings (Fig. 1). In the first phase, a multidisciplinary scientific committee was stablished, comprising experts specializing in medical oncology, hepatobiliary surgery, radiology, and nuclear medicine, all had experience managing patients with lmCRC using Y-90 TARE. Under the committee’s supervision, the objective and criteria for evidence review were determined through a comprehensive bibliographic search protocol for Y-90 TARE in managing lmCRC. The search encompasses the Medline database (PubMed) and international congresses communications. The PubMed search was limited to the last 5 years (April 2017–April 2022), with conference communications restricted to the previous 2 years. Editorial letters or case reports were excluded. In the second phase, data extraction was carried out by addressing an initial set of 34 preliminary questions, subsequently consolidated into 10 final questions to facilitate the understanding and provide recommendations for this document (Table 1). Based on the identified publications, the information was analyzed and distributed accordingly. All authors contributed to developing the guidelines, providing a critical review of the evidence, and finalizing the proposed recommendations in this guide. In situations where there was no evidence but a high level of agreement among panel members, informal consensus was reached.

**Table 1:** Questions addressing Y-90 TARE recommendations in lmCRC

Process	Questions
General issues	1. Which members should be part of the multidisciplinary tumor committee (MTC) for the selection of patients who are candidates for Y-90 TARE?
	2. What are the indications for treatment with Y-90 TARE?
	3. What are the contraindications for Y-90 TARE?
Previous process	4. Are there biomarkers that can predict candidate patients for Y-90 TARE treatment?
	5. What are the preintervention tests with Y-90 TARE?
	6. What is the evidence for Y-90 TARE treatment in lmCRC?
Procedure	7. What is the standard procedure for Y-90 TARE?
Post-intervention follow-up	8. What is the potential toxicity of Y-90 TARE, and how is it handled?
	9. What parameters are used for the evaluation of Y-90 TARE response?
	10. How does Y-90 TARE impact the quality of life of the patient with lmCRC?

## Results and discussion

### Characteristics and evaluation of the information

The identified publications included guidelines, systematic reviews and/or meta-analyses, clinical trials, and observational studies. Considering the robustness and quality of the information, national and international clinical guidelines served as reference documents for lmCRC patient management. These included the most recent versions of the European Society for Medical Oncology (ESMO) 2022 [5], the European Association of Nuclear Medicine (EANM) 2022 [3], the National Comprehensive Cancer Network (NCCN) v1.2022 [6], the Spanish multidisciplinary consensus (SMC) 2020 [7] from scientific societies (including the Spanish Society of Medical Oncology [SEOM], the Spanish Association of Surgeons [AEC], the Spanish Society of Radiation Oncology [SEOR], the Spanish Society of Vascular and Interventional Radiology [SERVEI] and the Spanish Society of Nuclear Medicine and Molecular Imaging [SEMNIM]), the SEOM clinical guideline for the diagnosis and treatment of lmCRC [8], and the consensus of the EANM dosimetry committee [9]. Subsequently, the analysis prioritized phase III clinical trials and prospective, ambispective, and retrospective observational studies. The selection of studies considered aspects related to the sample size, the stage of the study population, and relevance to clinical practice that is adaptable to the Spanish context.

### Recommendations

#### Question 1: Which members should be part of the multidisciplinary tumor committee (MTC) to select patients who are candidates for Y-90 TARE?

***Recommendation 1.1. MTC members evaluating candidates for treatment with Y-90 TARE should include at least surgeons specializing in hepatobiliary surgery, medical oncology and radiation therapy, vascular and interventional radiology, and nuclear medicine. It is also recommended to include radiopharmacists and radiophysicists.***

The management strategies for patients with lmCRC are constantly evolving, necessitating a multidisciplinary approach in the decision-making process for intervention with Y-90 TARE. This approach involves a group of experts who regularly convene to ensure proper patient selection, aiming to achieve maximum clinical benefit with a favorable toxicity profile. The significance of this multidisciplinary approach is emphasized in national [7] and international guidelines, such as ESMO 2022 [5] and EANM 2022 [3]. Similarly, publications reflecting clinical practice in Spain highlight the importance of a multidisciplinary approach in decision-making for the Y-90 TARE treatment strategy [10–13].

#### Question 2: What are the indications for treatment with Y-90 TARE for patients with lmCRC?

***Recommendation 2.1. In routine clinical practice, the use of Y-90 TARE in lmCRC is intended for patients with predominantly hepatic disease that is refractory or intolerant to chemotherapy. In a subset of patients limited to clinical trials setting, Y-90 TARE is used in the early stages of treatment (e.g., in patients who are potential candidates for resection but would have a small remaining liver volume [future liver remnant, FLR] post-resection).***

Guidelines support using Y-90 TARE therapy for patients who are not optimally resectable, have not responded to available chemotherapy agents, and have limited liver disease [3, 5–7]. These recommendation correspond to level III-B (ESMO 2022 [5]) and category 2A (NCCN [6]). The ESMO 2022 [5] further suggest considering its use as consolidation treatment in previous lines, although limited to clinical trial environments [5]. The SMC 2020 [7] also recommended therapy for unresectable patients who are refractory to systemic chemotherapy and for neoadjuvant treatment in technically inoperable patients to facilitate subsequent resection of metastasis.

***Recommendation 2.2. The clinical selection criteria for Y-90 TARE should be include laboratory examinations, such as blood count, coagulation, liver, and renal profile. Prognostic markers such as carcinoembryonic antigen (CEA) should also be evaluated. In addition, it is recommended to evaluate the patient’s treatment history, including previous surgeries and local treatments, such as chemoembolization (TACE) and/or local ablation techniques.***

**Fig. 2:**
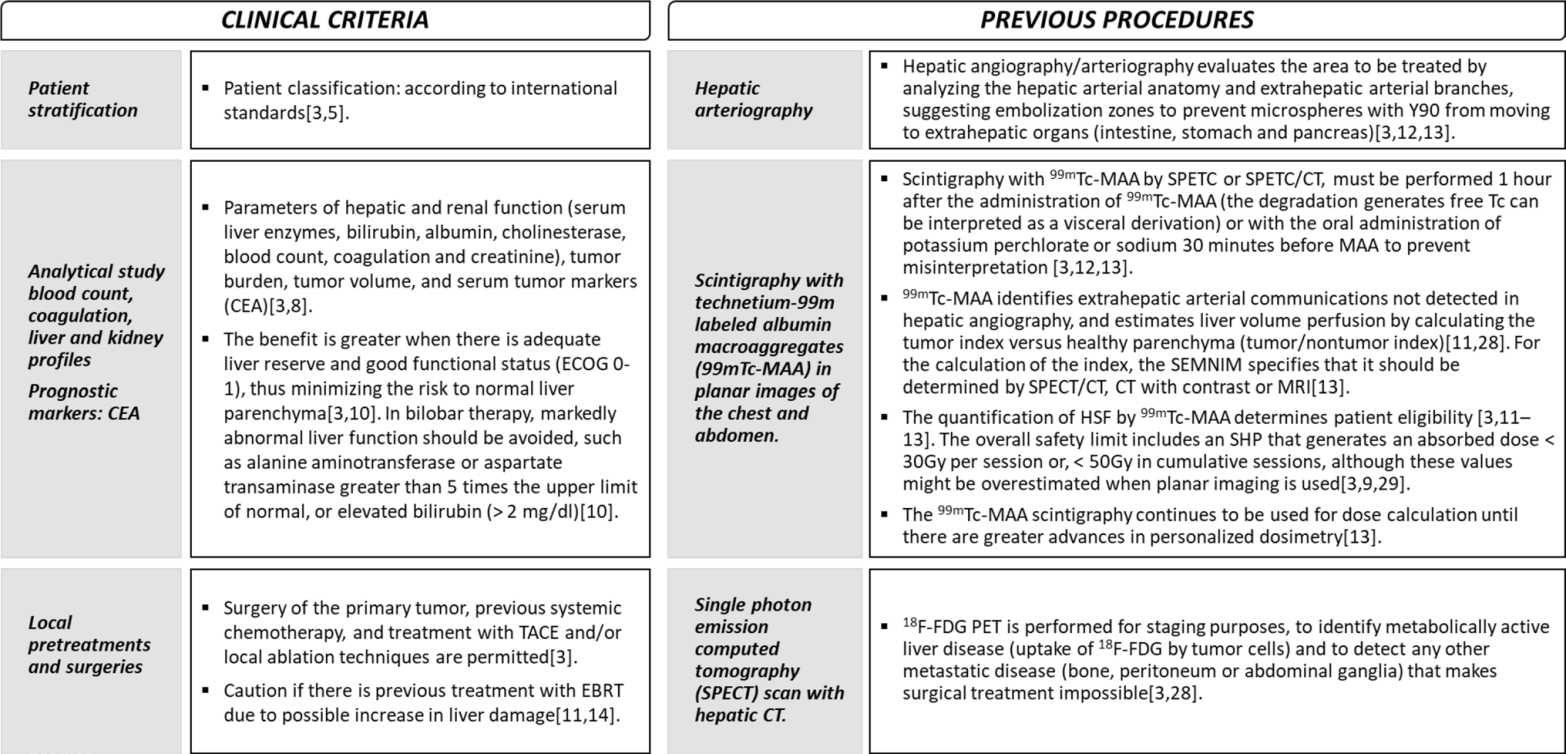
Clinical, laboratory, and imaging evaluations prior to the procedure. ^*18*^*F-FDG* 18Fluor-fluorodeoxyglucose; *CEA* carcinoembryonic antigen; *CT* computed tomography; *EBRT* external beam radiotherapy; *HSF* hepatopulmonary shunt fraction; *MRI* magnetic resonance imaging; *PET* positron emission tomography; *SEMNIM* Spanish Society of Nuclear Medicine and Molecular Imaging; *SPECT* single-photon emission computed tomography; *TACE* transarterial chemoembolization

The pre-treatment clinical evaluation of patients undergoing Y-90 TARE begins with stratification based on international standards, followed by a series of laboratory examinations conducted within 30 days prior to the intervention [3, 8, 10] (Fig. 2). It is also essential to consider the number of previous treatments lines and the specific treatments received prior to Y-90 TARE intervention. This may include surgery of the primary tumor, previous systemic chemotherapy, treatment with TACE, as well as local ablation technique are allowed [3].

#### Question 3: What are the contraindications for Y-90 TARE?

***Recommendation 3.1. Absolute clinical criteria contraindicating treatment with Y-90 TARE include circumstances of pregnancy, lactation, life expectancy < 3 months, clinical hepatic impairment, and disseminated extrahepatic disease (EHD). Relative clinical criteria that potentially contraindicate Y-90 TARE treatment include elevated Child–Pugh score (> 7 with an increased likelihood of hepatic decompensation), elevated intrahepatic (> 50–70% replacement of liver parenchyma by the tumor) or extrahepatic tumor burden, acute or severe chronic renal failure (creatinine clearance < 30 ml/min), and previous external beam radiation therapy (EBRT).***

The EANM 2022 [3] provides a differentiation between absolute and relative contraindications for the use of Y-90 TARE. In addition, Y-90 TARE therapy is safer in cases, where the patient has had limited hepatic exposure to EBRT, as previous liver exposure to EBRT can exacerbate liver toxicity following Y-90 TARE treatment. The cumulative thresholds of liver tolerance for combined radiation exposure are still unknown [11, 14].

***Recommendation 3.2. Disseminated EHD is contraindicated in Y-90 TARE treatment. However, when there is limited EHD (defined as the presence of metastases in hilar lymph nodes ≤ 2 cm and the presence of up to 5 lung nodules ≤ 1 cm), TARE treatment may be considered.***

Although EHD is associated with lower overall survival (OS) [15], patients with limited EHD might benefit from Y-90 TARE [3, 5]. In the context of lmCRC, the definition of limited EHD considers a threshold ≤ 5 nodules, either pulmonary and/or lymph nodes in the same region (SIRFLOX [16]), or metastases susceptible to future treatment (FOXFIRE [17]) [3]. The National Institute of the Netherlands defines acceptable limited EHD as the presence of a single lymph node metastasis (same region; diameter ≤ 2 cm) and/or lung metastases (≤ 5 metastases; diameter < 1 cm) and when the prognosis is determined by liver disease [18, 19].

#### Question 4. Are there biomarkers that can predict candidate patients for Y-90 TARE treatment?

***Recommendation 4.1. Currently, no prospective validated biomarkers allow the selection of candidate patients for Y-90 TARE. The results of subgroup analyses suggest a better response in patients with an absence of baseline EHD, low tumor burden, CEA levels ≤ 20 ng/ml, a good Karnofsky index, ECOG, and hematological status.***

Genetic biomarkers have been extensively studied in CRC, including in the context of Y-90 TARE. KRAS mutations, found in approximately 47% of patients with lmCRC treated with Y-90 TARE, have been identified as potential biomarkers associated with increased progression-free survival (PFS) [20, 21]. However, these findings are inconsistent with previous studies that reported a better response to Y-90 TARE in patients with native KRAS than those with mutant KRAS [22, 23]. On the other hand, no predictive association has been found between BRAF V600E mutations, elevated microsatellite instability, and the outcome of Y-90 TARE [11].

In relation to clinical and metabolic biomarkers, the retrospective study by Kurilova et al. [24] in the Netherlands analyzed multiple factors related to Y-90 TARE. It develops a nomogram consisting of six parameters (presence of EHD, number of EHD locations, CEA levels, albumin, alanine aminotransferase, and tumor differentiation levels) to predict OS. They also identified maximum standardized uptake value (SUVmax) as the only predictor of hepatic PFS. Damm et al. [25] in Germany found associations between OS outcomes and a scoring system comprising three factors (tumor burden > 20%, Karnofsky index < 80%, and CEA level > 130 ng/ml or CA19.9 > 200 U/ml). In the prospective observational study by Triviño-Ibáñez et al. [10] (Spain), factors associated with increased OS were primary tumor resection, absence of KRAS mutations, and hematological status (hemoglobin, neutrophil–lymphocytosis and platelet–lymphocyte ratio). The influence of the laterality of the primary tumor on the response to treatment with Y-90 TARE requires, further research to establish its association as part of the patient selection criteria.

Finally, imaging biomarkers, such as positron emission tomography (PET) combined with computed tomography (CT) with 18fluorodeoxyglucose (^18^F-FDG), before and after the intervention can also predict OS and PFS.A significant decrease (> 30%) in the uptake of ^18^F-FDG in liver metastases after the procedure could be associated with increased survival [26].

#### Question 5. What are the preintervention tests with Y-90 TARE?

***Recommendation 5.1. Procedures for patient selection include hepatic imaging and scintigraphy with technetium-99m labelled albumin macroaggregates (*** ^***99m***^ ***Tc-MAA), performing planar images of the chest and abdomen, and single-photon emission computed tomography (SPECT) with hepatic CT. Hepatic arteriography determines the hepatic arterial anatomy of the treated area. It allows for correction (embolization) of the regions that can cause future complications (possible leakage of the microspheres to other organs).*** ^***99m***^ ***Tc-MAA scintigraphy using SPECT or SPECT/CT allows us to simulate and anticipate the behavior of microspheres with Y-90, which is relevant for the planning of the procedure. This planning also allows for selecting candidate patients by detecting any other derivation of extrahepatic arterial communications not detected by hepatic angiography. In addition, it allows for the calculation of personalized dosimetry using a pulmonary shunt or hepatopulmonary shunt (HPS) and the tumor-to-healthy liver parenchyma uptake ratio.***

In addition to clinical evaluations, a functional imaging evaluation is crucial prior to and during the procedure with Y-90 TARE. Previous imaging tests, performed 30 days before the procedure, assess the feasibility of intervention with Y-90 TARE and serve multiple objectives, including staging, mapping of hepatic vascularization, simulation of microsphere distribution, and determination of tumor volume calculation [3, 11–13]. Contrast imaging techniques (CT, SPECT, and magnetic resonance imaging [MRI]) are employed for the various tests outlined in Fig. 2 [3, 11–13, 27–30].

***Recommendation 5.2. Imaging tests to assess the resectability of metastases should first include abdominal/pelvic and thoracic CT. If there is uncertainty and depending on the location of the metastases and therapeutic possibilities, a second method, should be progressively included, such as MRI, PET/CT, and abdominal ultrasound.***

Imaging techniques used to evaluate the secondary resectability of liver metastases include CT and MRI, while hepatic ultrasound and contrast ultrasound may be used to minimize radiation exposure [5]. Abdominal/pelvic and thoracic CT are commonly used to measure visible lesions with negative margins and sufficient FLR size [5, 31]. The MRI is used in lesions smaller than 10 mm in diameter, because it is more sensitive than CT in these cases [5]. For patients at high risk for local recurrence of EHD, CT, and PET are recommended [5].

#### Question 6. What is the evidence for Y-90 TARE treatment in lmCRC?

***Recommendation 6.1. In patients with potentially resectable liver metastases and oligometastatic disease, clinical evidence supports an indication for 90-Y TARE as a bridge therapy to surgery.***

In assessing the resectability of lmCRC, the MTC will define the primary tumor status in cases of involving synchronous metastases and/or oligometastatic disease [5]. Oligometastatic disease is defined as having ≤ 3 visceral or lymphoganglionary lesions without multiple metastases in the bones and brain (except for a single bone lesion attached to ≤ 2 resectable liver metastases) [5]. In oligometastatic disease, Y-90 TARE can allow for subsequent complete R0 resection and, in turn, could be potentially curative in 20–50% of cases [5]. The lmCRC is considered resectable when complete R0 resection of lesions will preserve the two adjacent liver segments, vascular inflow and outflow, adequate biliary drainage, and an FLR ≥ 20% [11, 32]. In addition, lmCRC should only be considered unresectable after 2–4 months of treatment with optimal systemic therapy [11].

***Recommendation 6.2. Regarding the clinical evidence that exists for the indication of Y-90 TARE in the first-line treatment of unresectable lmCRC*** **,** ***the results of the three phase III clinical trials FOXFIRE, SIRFOX and FOXFIRE-Global reported that the addition of Y-90 TARE to the systemic chemotherapy FOLFOX first-line treatment in patients with lmCRC is not recommended.***

The pooled results from the phase III clinical studies FOXFIRE, SIRFLOX, and FOXFIRE-Global, which included patients with similar first-line eligibility criteria in lmCRC, analyzed a total of 1103 patients receiving Y-90 TARE plus chemotherapy (*n* = 554) vs chemotherapy (*n* = 549). With a median follow-up of 43.3 months, the median OS of combination therapy was 22.6 vs 23.3 months with FOLFOX (5-fluorouracil, leucovorin, and oxaliplatin) (HR 1.04; 95% CI 0.90–1.19, *p* = 0.61) [33]. The median PFS in the combination therapy was 11.0 months (95% CI 10.2–11.8) vs 10.3 months with FOLFOX (HR 0.90; 95% CI 0.79–1.02, *p* = 0.11) [33]. The objective response rate (ORR) was higher with combination therapy (72% vs 63%) (pooled OR 1.52; 95% CI 1.18–1.96, *p* = 0.0012). Furthermore, the hepatic ORR was also higher in combination therapy vs FOLFOX (pooled OR 1.78; 95% CI 1.37–2.31, *p* < 0.0001). These findings suggest that the addition of Y-90 TARE to first-line FOLFOX systemic chemotherapy is not recommended [33].

***Recommendation 6.3. Second-line treatment with Y-90 TARE for unresectable lmCRC is supported by clinical evidence from the phase III EPOCH clinical trial and observational studies.***

The clinical evidence supporting the indication of Y-90 TARE as a second-line treatment for unresectable lmCRC is derived from the phase III EPOCH study, which employed glass microspheres [20, 21]. This study compared the addition of Y-90 TARE to second-line systemic chemotherapy in patients with lmCRC who progressed from a first-line treatment based on oxaliplatin or irinotecan; which also includes biological agents [20]. The study demonstrated improved PFS and hepatic PFS outcomes with combination therapy using Y-90 TARE [20].

With a median follow-up of 36.0 and 42.3 months for the combination therapy and control group, respectively, the median PFS was 8.0 (Y-90 TARE plus chemotherapy) vs 7.2 (chemotherapy) months (HR = 0.69; 95% CI 0.54–0.88; one-sided *p* = 0.0013). The median hepatic PFS was 9.1 (Y-90 TARE plus chemotherapy) and 7.2 (chemotherapy) months (HR = 0.59; 95% CI 0.46–0.77; one-sided *p* < 0.0001) [20]. The ORR for combination therapy was 34.0% (95% CI 28.0–40.5), and it was 21.1% (95% CI 16.2–27.1; one-sided *p* = 0.0019) for chemotherapy [20]. The median OS was 14.0 (95% CI 11.8–15.5) months for combination therapy and 14.4 (95% CI 12.8–16.4; one-sided *p* = 0.7229) months for chemotherapy (HR = 1.07; 95% CI 0.86–1.32) [20]. Toxicity was higher with Y-90 TARE (grade ≥ 3 adverse events [AEs]: 68.4% vs 49.3%) [20]. The results showed that the addition of Y-90 TARE to second-line systemic chemotherapy prolongs PFS and hepatic PFS in lmCRC.

Retrospective observational studies have investigated the use of Y-90 TARE as savage therapy in patients who were refractory to at least a first line of systemic chemotherapy. These studies reported median OS outcomes of 12.0 months in the study by Saxena et al.* 2015* (*n* = 159; *N* = 302) [34], and 13.2 months in the study by Kennedy et al*.* 2017 (*n* = 206; *N* = 606) [35].

***Recommendation 6.4. Clinical evidence supports using Y-90 TARE in highly selected patients with hepatic-resistant or chemotherapy–refractory hepatic predominant lmCRC.***

Treatment with Y-90 TARE has traditionally been administered as salvage therapy for lmCRC after failure of 2 or more previous lines. Unicentric (*N* = 104–302) and multicentric (*N* = 531–606) retrospective studies reflect OS results ranging from 10.0 to 10.6 months [15, 34–38]. Systematic reviews evaluating Y-90 TARE in patients with unresectable and refractory lmCRC chemotherapy (*N* = 979 [39]; *N* = 901 [40]) reflect OS data up to 12 months. In patients with chemotherapy–refractory lmCRC, improved time to progression has been observed when TARE is combined with 5-FU compared to 5-FU monotherapy [41].

***Recommendation 6.5. The optimal time interval to consider any surgical intervention after the procedure with Y-90 TARE will depend on the time spent to obtain the maximum benefit (which may vary between 2 and 10 months).***

The available data derived from a small number of patients with HCC (*n* = 5), lmCRC (*n* = 5) and neuroendocrine tumors (*n* = 2), reported mean time from intervention with Y-90 TARE to resection of 322 days (range 195–703) [42]. According to an analysis by Berry et al. [43], waiting at least 8 weeks after an intervention with Y-90 TARE until surgery is recommended; however, the optimal time will depend on achieving the maximum benefit that TARE can present, which is between 3 and 6 months.

#### Question 7. What is the standard procedure for Y-90 TARE?

***Recommendation 7.1. The relationship between the dose administered and the average dose absorbed by the tumor in lmCRC treated with Y-90 TARE is under continuous study, and its optimization is challenging. There is a relationship between the absorbed dose and the response rate, so the recommended dose would be the maximum possible, safeguarding the dose absorbed by the lungs and healthy liver tissue.***

Dosimetry calculation is an essential parameter in Y-90 TARE due to the different distributions of microsphere activity between healthy liver parenchyma and tumor tissue [12]. The dose–response relationship varies depending on the types of microspheres used (resin and glass) [9, 44].

Dosimetry can be performed by three methodologies: single-compartment, multiple-compartment, and voxel-based [3]. In the single-compartment model, a mean dose is administered to the entire perfused hepatic volume, considering a uniform distribution of microspheres without differentiation between the tumor and the normal liver parenchyma, presenting as a limitation the administration of low doses or potential overdoses [3]. The multi-compartment or partition model evaluates the average dose in each compartment (tumor, normal liver and lung tissue), allowing for maximizing the dose to the tumor tissue while staying within toxicity thresholds for other compartments; this model is limited by the heterogeneity of the dose distribution in each compartment [3]. The third method, based on voxels, enables the estimation of dose gradients and non-homogeneities on a small spatial scale [3]. The measurements for dosimetry include ^99m^Tc-MAA SPECT/CT with or without attenuation correction and PET/CT imaging [9].

Posttreatment imaging techniques include PET, planar scintigraphy, and bremsstrahlung SPECT/CT [3]. PET/CT provides superior quantification and spatial resolution compared to the bremsstrahlung SPECT/CT technique [7]. Planar imaging helps identify unanticipated lung bypasses and investigate complications using 3D imaging [3, 45, 46].

***Recommendation 7.2. The discontinuation of systemic chemotherapy treatment at least 2 weeks and up to 14 weeks prior to the beginning of Y-90 TARE treatment is recommended.***

Retrospective studies that report data from routine clinical practice, suggests that discontinuation of systemic chemotherapy typically occurs between 2 [47] and 4 [48] weeks prior to the intervention with Y-90 TARE. According to the Spanish multidisciplinary review, if there is evidence of low uptake of ^99m^Tc-MAA in the tumor tissue an 8-week interval following the suspension of angiogenic drugs (bevacizumab and sunitinib), it is recommended to wait an additional 6 weeks before proceeding with Y-90 TARE to achieve greater efficacy [13]. This extended interval is advised to enhance the efficacy of Y-90 TARE treatment.

***Recommendation 7.3. Types of hepatic approaches with Y-90 TARE include radiation segmentectomy, lobectomy (with and without portal and/or hepatic vein tumor thrombus [PVT]), and treatment of both lobes (bilobar). Radiation segmentectomy is considered an exploratory strategy aimed at patients with oligometastatic disease who are not amenable to curative treatment (percutaneous ablation or resection).***

Several clinical scenarios can arise during the planning of Y-90 TARE, including the following [3]:

(i) Radiation segmentectomy in oligometastatic lmCRC, where the liver disease is limited to ≤ 2 segments, it can generate improved response rates, higher PFS, and no increased risk of liver damage [49, 50]. Several retrospective studies with small cohorts have reported favorable results in lmCRC patients (Meier et al. [50], Padia et al. [51], Kurilova et al. [52]).
(ii) Radiation lobectomy is an option in lmCRC cases with unilobar diseases or cases, where hypertrophy of the contralateral lobe can be induced. This allow patients to undergo resection with curative intent (downstaging strategy), with evidence using glass and resin microspheres [44, 53]. In cases where lobar disease is not amenable to curative surgery, Y-90 TARE can be used as a palliative treatment option [3]. It is important to note that evidence in cases of HCC suggests that Y-90 TARE does not increase liver toxicity only in patients with lobar disease and PTV [54].
(iii) Bilobar radiation can be performed as a single or sequential procedure. However, it should be noted that, while high doses absorbed by tumors correlate with improved response, doses absorbed by functional liver parenchyma increase the risk of radioembolization-induced liver disease (REILD) [3].

***Recommendation 7.4. In bilobar disease, sequential treatment is preferred, because it allows for recovery of the treated lobe, and dosimetry for the second treatment can be adjusted based on the first one. The interval between two lobar treatments ranges from doing it on the same day to 90 days.***

In a sequential bilobar treatment, the dose obtained from the first procedure can be adjusted for the second intervention to optimize treatment outcomes [3]. Depending on the patient's liver function, the intervention in both lobes can be performed in one or two sessions [55]. The time intervals between treatments may vary depending on the types of microspheres. For sequential lobar interventions with glass microspheres the gaps can range from ≤ 30, 40, and 60 days, or even up to 90 days, mainly when there is a poor balance between dosimetry and patient characteristics, such as liver cirrhosis, liver volume < 1.5 L, or elevated bilirubin [3, 55–58].

***Recommendation 7.5. Retreatment with Y-90 TARE has been reported to be noncritical, although ongoing assessment of cumulative absorbed doses to the lung parenchyma is recommended.***

Retreatment with Y-90 TARE in lmCRC has limited and non-specific evidence, as stated in the EANM 2022 guidelines [3]. The decision for retreatment depends on the liver’s regenerative capacity, with an interval of at ≥ 3 months between treatments [3]. Retreatment with Y-90 TARE is a feasible strategy, particularly in patients who have responded to the first treatment [3, 59]. It is noteworthy that retreatment with Y-90 TARE appears to be less critical than interventions with Y-90 TARE after EBRT [3, 59]. In patients with EHD, available data regarding reintervention with Y-90 TARE indicate an average lobar repeat of 2.6-fold, with an incidence of toxicity at 17% [60].

#### Question 8. What is the potential toxicity of Y-90 TARE, and how is it handled?

***Recommendation 8.1. Y-90 TARE can cause mild–moderate to severe AEs (REILD and nontarget organ irradiation).***

Common mild or moderate AEs (incidence > 10%) include fatigue, abdominal pain, nausea, fever or chills, transient elevation of liver enzymes, a transient decrease in lymphocytes, and constitutional symptoms (asthenia, weight loss, and anorexia) [3, 27]. Severe AEs, with an incidence < 5%, include unwanted target irradiation (radiation gastritis, gastrointestinal ulceration, upper gastrointestinal bleeding, pancreatitis, and radiation pneumonitis) and REILD (jaundiced ascites, hepatomegaly, and increased ratio of transaminases to elevated bilirubin), which occurs between 2 and 6 months after treatment and without disease progression [3, 11]. The risk of REILD increases when patients have been previously treated with chemotherapy [11, 61] or in cases in which more than two procedures are performed in a single lobe [59].

The EANM 2022 [3] recommends considering prophylactic antibiotics in patients with a history of biliary intervention (increased risk of cholangitis or abscess), although direct evidence is only related to TACE.

***Recommendation 8.2. Adequate biochemical monitoring is relevant to control toxicity with Y-90 TARE.***

Monitoring potential AEs following Y-90 TARE should be consider pretreatment liver function and treatment protocols. Non-dosimetric methods (body surface area-based methods and compartment models) may result in insufficient or overtreatment; notably, an absorbed dose of functional liver tissue ≥ 30 Gy can lead to radiation hepatitis [3, 12]. Therefore, careful consideration should be given to dose planning and optimization.

After Y-90 TARE, regular follow-up at 2 or 4 weeks with biochemical monitoring and clinical evaluations is recommended. In patients who have undergone ≥ 3 previous chemotherapy treatments, grade 1–2 AEs are common and usually resolve without intervention [39].

#### Question 9. What parameters are used for the evaluation of Y-90 TARE response?

***Recommendation 9.1. Radiological changes induced by Y-90 TARE are assessed using Response Evaluation Criteria in Solid Tumors (RECIST). Imaging techniques commonly used include CT or MRI and FDG–PET.***

In assessing treatment response to Y-90 TARE in patients with ImCRC, the RECIST 1.1 criteria have been commonly used [62]. These criteria were used to determine the response to Y-90 TARE treatment in patients with lmCRC providing a correlation between the treatment response and OS [20, 33]. However, the modified RECIST criteria (mRECIST) do not demonstrate the same correlation [3, 63]. In lmCRC, response to Y-90 TARE has been assessed using several criteria (RECIST 1.1, tumor attenuation, Choi and PET of the European Organization for Research, and Treatment of Cancer [PET–EORTC]); these were compared in terms of their correlation with PFS, finding RECIST 1.1 criteria to have a low sensitivity for detecting metabolic responses compared to PET–EORTC criteria [64].

***Recommendation 9.2. Response times to Y-90 TARE treatment are variable, depending on the type of imaging test used and what is considered a response measure. To assess early response within 1 to 3 months*** **,** ^***18F***^ ***FDG PET/CT can be used, while diffusion-weighted imaging or changes in metastases size could be observed for 3 to 4 months.***

In the context of lmCRC, ^18F^FDG–PET/CT has demonstrated the ability to detect functional changes that precede structural changes, allowing for early assessment of treatment response (6–8 weeks) compared to conventional imaging [3]. The optimal response assessment time after Y-90 TARE in lmCRC remains uncertain, with different studies suggesting assessment at 1, 3, and 6 months after treatment and depending on the measurement of response, either by controlling the disease or by decreasing the tumor volume [3, 65].

Studies have shown that early follow-up assessments at 1 and 3 months using ^18F^FDG PET/CT are more sensitive and accurate predicting OS compared to assessments based solely on tumor size reduction determined by MRI. This suggest that metabolic response, as detected by ^18F^FDG PET/CT, may precede anatomical response, with earlier changes in metabolic activity indicative of treatment efficacy [66].

#### Question 10. How does Y-90 TARE impact the quality of life of patients with lmCRC?

***Recommendation 10.1. In clinical practice, the most appropriate instruments for measuring patients' health-related quality of life (HRQoL) with lmCRC treated with Y-90 TARE include the generic questionnaires EQ-5D-3L and the EORTC QLQ-C30. The colorectal liver cancer module (EORTC QLQ-CML21) and the functional assessment of cancer therapy against colorectal cancer (FACT-C) questionnaire can be used as more specific alternatives.***

Clinical trials evaluating Y-90 TARE in patients with lmCRC, either in first-line [67] and second-line [21] treatment with associated with systemic chemotherapy, have utilized various instruments to assess HRQoL.

The EQ-5D-3L questionnaire has been commonly used as a generic instrument to assess HRQoL in these studies [68, 69]. It provides a standardized measure of health status across different conditions and treatment modalities. The EORTC QLQ-C30 questionnaire, a widely used cancer-specific HRQoL instrument, has also been employed to evaluate the impact of Y-90 TARE on various aspects of a patients' well-being [70]. In addition, the EORTC QLQ–LMC21 module targets explicitly targets the assessment of HRQoL in patients with colorectal liver metastases, providing disease-specific information related to liver cancer [71]. The FACT-C questionnaire, which is a combination of the Functional Assessment of Cancer Therapy-General (FACT-G) questionnaire and the colorectal cancer-specific subscale (CRC subscale), has been utilized to assess HRQoL in lmCRC patients undergoing Y-90 TARE [72]. A recent prospective phase IV study (PROACTIF) [73] used the FACT-Hep questionnaire [74], which is an instrument for the functional evaluation of the treatment of hepatobiliary cancer (liver, bile ducts, and pancreas). This questionnaire comprehensively evaluates the functional status and well-being of patients with hepatobiliary tumors.

***Recommendation 10.2 The evidence identified on Y-90 TARE therapy and its impact on the quality of life of patients with lmCRC has been determined in mixed cohorts of patients or in first-line treatment. The results show that Y-90 TARE maintains the patient's HRQOL.***

In phase III clinical trials (FOXFIRE, SIRFLOX and FOXFIRE GLOBAL) involving patients with lmCRC (*N* = 1103), HRQoL was assessed using various instruments. The results showed that HRQoL decreased in the first 3 months after treatment in patients treated with first-line therapy, but this decline was not considered clinically significant [67]. Subsequent HRQoL evaluations conducted annually over a 2 years of follow-up and at the time of disease progression did not reveal a clinically significant decline either [67].

Prospective studies evaluating HRQoL using the EORTC QLQ-C30 questionnaire in mixed cohorts, including patients with lmCRC, have reported similar findings. These studies conducted in Europe and France showed that Y-90 TARE did not significantly impact the patients’ HRQoL [75, 76]. This suggests that Y-90 TARE treatment is generally well-tolerated and does not substantially deteriorate HRQoL in patients with lmCRC.

## Data Availability

Not applicable.

## Abbreviations

^18^F-FDG: ^18^Fluor-fluorodeoxyglucose
^99m^Tc-MAA: Technetium-99m labelled albumin macroaggregates
AE: Adverse event
AEC: Spanish Association of Surgeons
CRC: Colorectal cancer
CEA: Carcinoembryonic antigen
CIRSE: Cardiovascular and Interventional Radiological Society of Europe
CT: Computed tomography
PVT: Portal and/or hepatic vein tumor thrombus
EANM: European Association of Nuclear Medicine
EBRT: External beam radiotherapy
ECIO: European Conference on Interventional Oncology
ECR: European Congress of Radiology
ESMO GI: World Congress on Gastrointestinal Cancer of European Society for Medical Oncology
FLR: Future liver remnant
FOLFOX: 5-Fluorouracil, leucovorin and oxaliplatin
GI ASCO: Gastrointestinal Cancers Symposium of American Society of Clinical Oncology
HCC: Hepatocellular carcinoma
HRQoL: Health-related quality of life
lmCRC: Liver metastases from colorectal cancer
MRI: Magnetic resonance imaging
MTC: Multidisciplinary tumour committee
NCCN: National Comprehensive Cancer Network
ORR: Objective response rate
OS: Overall survival
PET: Positron emission tomography
PFS: Progression-free survival
RECIST: Response evaluation criteria in solid tumors
REILD: Radioembolization-induced liver disease
SEMNIM: Spanish Society of Nuclear Medicine and Molecular Imaging
SEOM: Spanish Society of Medical Oncology
SEOR: Spanish Society of Radiation Oncology
SERVEI: Spanish Society of Vascular and Interventional Radiology
SMC: Spanish multidisciplinary consensus
HPS: Hepatopulmonary shunt
SIO: Society of Interventional Oncology
SNMMI: Society of Nuclear Medicine and Molecular Imaging
SPECT: Single photon emission computed tomography
TACE: Transarterial chemoembolization
Y-90 TARE: Yttrium-90 transarterial radioembolization

## Declarations

### Disclaimer of recommendations

The expert panel provides these recommendations to assist professionals in clinical decision-making. Since there is a continuous updating of the treatments, it cannot be assured that the information contained in this document is complete. This guide provides information that is not intended to replace the clinical judgements of health professionals due to individual variability among patients. Therefore, the use of this information is voluntary.
